# The use of complementary and alternative medicine in a multi-ethnic Asian population: results from the 2016 Singapore Mental Health Study

**DOI:** 10.1186/s12906-020-2843-7

**Published:** 2020-02-13

**Authors:** Vanessa Seet, Edimansyah Abdin, Janhavi A. Vaingankar, Shazana Shahwan, Sherilyn Chang, Bernard Lee, Siow Ann Chong, Mythily Subramaniam

**Affiliations:** 1 Research Division, Institute of Mental Health, Singapore, Singapore; 2 Ministry of Health, Singapore, Singapore; 3 Lee Kong Chian School of Medicine, Singapore, Singapore

**Keywords:** Ethnic differences, Complementary and alternative medicine, Patterns of use, Mental illness

## Abstract

**Background:**

This study seeks to investigate factors associated with using complementary and alternative medicine (CAM) for a mental illness among the three major ethnic groups (Chinese, Indians and Malays) in the general population of Singapore.

**Methods:**

Data from the 2016 Singapore Mental Health Study was used; responses from the “Services” section of the Composite International Diagnostic Interview version 3.0 (CIDI 3.0) administered during face-to-face household interviews with participants were analyzed to establish prevalence of CAM use among Singaporeans. Additionally, sociodemographic variables of interest were selected for sub-group regression analyses to yield correlates of CAM use among the three ethnic groups.

**Results:**

6.4% of Singaporeans used at least one form of CAM in the past 12 months for their mental illness. Malays reported using CAM the most, followed by Indians and Chinese. Sociodemographic variables such as education and employment were differently associated with CAM use among the ethnicities. Across all three ethnic groups, CAM users were more likely to report poorer mental health-related quality of life.

**Conclusion:**

Despite the significant differences in CAM use among Chinese, Malays and Indians, those who had a mental illness were significantly more likely to use CAM regardless of ethnicity. This highlights the need for communication between CAM practitioners and conventional mental healthcare providers for early referral when appropriate which would lead to improved healthcare delivery and better clinical outcomes.

## Background

According to the World Health Organization (WHO) [1], complementary or alternative medicine (CAM) refers to “a broad set of health care practices that are not part of that country’s own tradition or conventional medicine and are not fully integrated into the dominant health-care system.” The concept of CAM is not a new one, and the use of CAM for health promotion and the treatment of physical illness has been increasing worldwide over the years. Overall, the prevalence of all types of CAM use range from 9.8 to 76%, with the wide variation due to factors such as differences in the population characteristics, response rates, CAM definitions used, and study methodology [2, 3]. There is a slow but steady increase of 2 to 6 % of CAM use within a decade in Western countries such as the United States, United Kingdom, and Australia [4]. In East Asian countries, the 12-month prevalence of general CAM use is also high, with more than half the general population reported to be utilizing CAM in Malaysia, South Korea and Japan [5–7].

CAM has also been utilized for mental illness. In the US, significantly more people with moderate mental distress reported the use of CAM when compared with the general population [8]. In another study involving participants from 25 countries, CAM usage was reported by a total of 3.6% of respondents who met the criteria for a mental disorder as specified in the Diagnostic and Statistical Manual of Mental Disorders, Fourth Edition within the past 12 months, with increased prevalence in higher-income countries as opposed to lower-income countries [9].

There is also evidence showing the potential benefits of using certain CAM modalities, including exercise and herbal therapies, for mental illness in terms of illness management and health promotion [10, 11]. On the other hand, other modalities, such as biologically-based therapies, may produce general health complications [12, 13] which is compounded by the tendency for patients not to report their usage of CAM to their physicians [14]. With its growing global traction as a complementary avenue for managing health and illness, it is important to gain a better understanding of CAM and the characteristics associated with its use, and to assess the effects of using both CAM and conventional therapies for any possible adverse effects.

In general, while previous studies have found associations between CAM use and gender, education, income level and health status as well as ethnicity, the extent and direction of such associations between CAM use and these variables have not been consistently established across these studies [8, 15–17]. In Singapore, CAM use has been documented in studies which found differing rates of use among Singaporeans and different variables associated with its use [18, 19]. However, this does not provide a full picture of how CAM is used among Singapore residents, with their ethnic backgrounds as a potential influencing factor on CAM use. Moreover, there is a dearth of research on the use of CAM as an alternative mental health treatment resource for people in Singapore, with only one other study examining the factors influencing CAM use among older people with depression [18].

To bridge the research gap in this area of CAM use, this article examines the factors influencing the use of CAM for the treatment of mental illness in the general population of Singapore. Specifically, the article investigates variations in sociodemographic associations with CAM use among the different ethnic groups as well as the link between health-related quality of life and CAM use. We hypothesized that the associations between CAM use and selected sociodemographic variables differ among the three main ethnic groups in Singapore (Chinese, Malays and Indians), and that CAM use is associated with poorer health-related quality of life. This article aims to provide new insight into the prevalence and patterns of general CAM use for mental illness in the Singaporean population according to the major ethnicities in the country and address the gaps in current literature.

## Methods

### Sample

Data on the use of CAM was taken from the 2016 Singapore Mental Health Study (SMHS 2016), an epidemiological study that aimed to examine the prevalence of mental illness in the general Singaporean population [20]. The detailed methodology of the SMHS 2016, which followed that of the first SMHS conducted in 2010, has been described in previous articles [20, 21]. The SMHS 2016 used a disproportionate stratified sampling method with 16 strata and the estimates were weighted back to achieve nationally representative data of the adult Singapore resident population (consisting of Singaporean citizens and permanent residents). To achieve adequate sample size for reliability of sub-groups analysis, the three main ethnic groups in Singapore were sampled in equivalent proportions of about 30% each, such that Malays and Indians were over-sampled as opposed to following the population’s ethnic distribution. Respondents were randomly sampled from a national registry of Singaporean and permanent residents and interviewed face-to-face in their residences from 2016 to 2018 in English, Mandarin or Bahasa Melayu after indicating their consent to take part in the study. The survey was administered using computer assisted personal interviews (CAPI) by professional survey interviewers who had undergone training, evaluation and supervision by the Institute of Mental Health’s research team. Specifically, these interviewers were trained for 2 weeks on ethics and study methodology, assessed by trainers on proper interview administration, and their competency evaluated via mock interviews before proceeding with actual participant interviews. Additionally, they underwent observation and were given feedback by the research team and their firm supervisor for their first five interviews. Finally, regular de-briefings were conducted to ensure prompt resolution of issues identified by interviewers in the field. Overall, 6126 people were interviewed in the SMHS 2016, with a response rate of 69.5% among the eligible adults.

### Questionnaires

#### CIDI 3.0

The World Mental Health Composite International Diagnostic Interview (CIDI), version 3.0 [22], was the main survey instrument used in the SMHS 2016. The CIDI is a research diagnostic interview of reasonable reliability and validity [23] that was developed by WHO to assess mental disorder prevalence in different populations [24] and was used in the WHO World Mental Health Surveys [25]. The CIDI was used to establish the lifetime prevalence of major depressive disorder, dysthymia, bipolar disorder, generalized anxiety disorder, obsessive compulsive disorder, alcohol abuse and alcohol dependence among respondents as well as use of alternative services in relation to mental disorders. In the Services section of the CIDI, respondents answered a question regarding their use of alternative therapies, which in the context of Singapore, whose dominant health care practice is conventional medicine, refer to therapies not offered in primary care hospitals and clinics, and which had to be sought outside of these settings. They were given a list of predefined therapies to choose from, and answered “yes” or “no” to the question of whether they “used any of these therapies in the past 12 months for problems with [their] emotions or nerves or [their] use of alcohol or drugs.” Therapies listed as options included acupuncture, exercise or movement therapy, herbal therapy, prayer or other self-directed spiritual practices, and massage therapy among others, as well as an option for “other non-traditional remedy or therapy” not included in the predefined list. Table 3 presents a full list of the types of CAM therapies identified.

#### SF-12

The 12-item Short Form Health Survey (SF-12) was used as a measure of health-related quality of life [26]. The SF-12 is a commonly used 12-question subset of the 36-item Short Form Health Survey (SF-36) and is used in place of its longer version for easier and faster administration. Like the SF-36, the SF-12 comprises two components, the physical component and the mental component, both of which consist of a further four subscales each [26]. Physical functioning, role-physical, bodily pain and general health subscales make up the physical component, while the mental component is made up of vitality, social functioning, role-emotional and mental health subscales. The reliability and validity of the SF-12 as a self-administered instrument of health-related quality of life has been established in both the general population and in patients with mental illnesses [26–28].

#### Sociodemographic questionnaire

Respondents completed a questionnaire pertaining to their sociodemographic information. For ethnicity, respondents specified whether they were Chinese, Malay, Indian, or of other ethnic backgrounds. Other sociodemographic information collected included age (re-categorized as age groups 18–34, 35–49, 50–64, and 65 and older), gender (male and female), marital status (never married, married, divorced, and widowed), average monthly household income in Singapore dollars (below 2000, 2000 to 3999, 4000 to 5999, 6000 to 9999, and 10,000 and above), education level (primary, secondary, pre-university/junior college, vocational/ITE, diploma, and university), and employment status (employed, economically inactive - such as housewives, students and National Servicemen – and unemployed).

### Statistical analyses

All estimates were weighted to the Singapore resident population in 2014 to adjust for over-sampling and non-response, and post-stratified for age and ethnicity distributions. Binary variables (yes/no) were created to assess whether the criteria for diagnosis of any mental disorder included in the CIDI modules was met. For CAM use, the main outcomes of interest were: use of any form of CAM in the past 12 months and use of a specific therapy in the past 12 months. For both outcomes, binary variables (yes/no) were similarly created for CAM use versus no CAM use. Percentages were calculated for categorical variables, while means were calculated for continuous variables. 95% confidence intervals were calculated for all variables. Cross-tabulations and chi square tests were used to investigate differences in CAM use by sociodemographic variables and the presence of a mental disorder as determined by the CIDI, while independent-sample T-tests were used to examine differences in CAM use by SF-12 component scores. The prevalence of different modalities of CAM use by ethnicity was estimated for the general residential population of Singapore. Finally, multiple logistic regression was conducted to identify variables associated with CAM use by the different ethnicities, and multiple linear regression analyses were conducted to investigate the association between 12-month CAM use and health-related quality of life. A *p*-value of 0.05 was chosen as the cut-off level of statistical significance for the analyses. The Statistical Analysis Software package (SAS), version 9.3, was used for all analyses [29].

## Results

### Sample characteristics

In total, after removing missing data and non-responses to the question on CAM use, responses from 6117 participants were analyzed, which constituted a representative sample of the adult resident population in Singapore. The sample comprised 1780 Chinese, 1986 Malays, 1841 Indians, and 510 people of other ethnicities. Due to the small number and heterogeneity of people of other ethnic backgrounds, only the characteristics of the three main ethnic groups of Singapore were subsequently analyzed. Table 1 presents sociodemographic characteristics of Singapore residents by CAM use within the past 12 months. Overall, there were statistically significant differences in almost all variables except for income.

**Table 1:** Sociodemographic characteristics of respondents

	Total	CAM use in past 12 months (%)		No CAM use in past 12 months (%)		*p*
	*n*	Weighted %	95% CI	Weighted %	95% CI	
Ethnicity						0.0083
Chinese	1780	5.9	4.9–7.1	94.1	92.9–95.1	
Malay	1986	8.1	6.9–9.6	91.9	90.5–93.1	
Indian	1841	7.6	6.4–9.0	92.4	91.0–93.6	
Others	510	8.5	6.2–11.7	91.5	88.3–93.8	
Age						< 0.001
18–34	1704	9.2	7.5–11.2	90.8	88.8–92.5	
35–49	1496	7.8	6.1–10.0	92.2	90.0–93.9	
50–64	1623	3.9	2.8–5.5	96.1	94.5–97.2	
65+	1294	1.8	1.0–3.2	98.2	96.8–99.0	
Gender						0.0013
Male	3064	4.9	3.9–6.2	95.1	93.8–96.1	
Female	3053	7.8	6.6–9.3	92.2	90.7–93.4	
Marital status						< 0.001
Married	1541	4.8	3.9–6.0	95.2	96.1–94.0	
Never married	3839	10.1	8.2–12.2	90.0	87.8–91.8	
Divorced/separated	343	4.4	2.6–7.4	95.6	92.6–97.4	
Widowed	394	4.1	1.9–8.7	95.9	91.3–98.1	
Education						< 0.001
University	1455	8.1	6.4–10.3	91.9	89.7–93.6	
Diploma	1023	8.6	6.5–11.3	91.4	88.7–93.5	
Vocational/ITE	508	6.7	4.4–10.1	93.3	89.9–95.6	
Pre-U/JC	304	7.0	3.9–12.1	93.0	87.9–96.1	
Secondary	1643	5.4	4.0–7.2	94.6	92.8–96.0	
Pri and below	1184	1.8	1.0–3.3	98.2	96.7–99.0	
Employment						< 0.001
Employed	4053	6.7	5.7–7.9	93.3	92.1–94.3	
Economically inactive	1714	3.8	2.7–5.3	96.2	94.7–97.3	
Unemployed	349	13.7	8.9–20.4	86.4	79.6–91.1	
Monthly Household Income						0.0572
Below 2000	1142	4.7	3.2–6.8	95.3	93.3–96.8	
2000–3999	1331	7.6	5.7–10.1	92.4	89.9–94.3	
4000–5999	1112	5.2	3.7–7.3	94.8	92.7–96.3	
6000–9999	1002	8.5	6.4–11.2	91.5	88.8–93.7	
10,000 and above	860	7.6	5.5–10.4	92.4	89.7–94.5	
Any mental disorder						
Yes	844	17.0	13.7–21	83	79–86.3	< 0.001
No	5273	4.7	3.9–5.6	95.3	94.4–96.1	

SF-12 scores were calculated and analyzed separately as physical and mental component scores. The means and 95% confidence intervals for SF-12 scores by CAM use within the past 12 months are displayed in Table 2. In general, respondents who reported using CAM in the past 12 months were not significantly different from non-users in their physical component scores, with only Role-Physical and Bodily Pain sub-scores showing significant differences between the two groups. However, CAM users reported lower mental component scores than their non-user counterparts (*p* < 0.001), with all four mental component sub-scores being significantly lower for CAM users (*p* < 0.001).

**Table 2:** SF-12 scores by 12-month CAM use

	Used CAM in past 12 months		No CAM use in past 12 months			
	Mean	95% CI	Mean	95% CI	*p*	Cohen’s *d*
Physical component score (PCS-12)	52.8	51.8–53.8	53.5	53.3–53.7	0.206	0.100
Physical functioning	54.1	53.1–55.1	54.6	54.4–54.9	0.307	0.080
Role-physical	53.2	55.2–54.1	55.4	55.2–55.5	< 0.001	0.342
Bodily pain	52.0	50.7–53.3	54.9	54.6–55.1	< 0.001	0.370
General health	49.3	47.8–50.8	50.8	50.5–51.1	0.051	0.157
Mental component score (MCS-12)	51.3	50–52.7	55.9	55.7–56.1	< 0.001	0.588
Vitality	54.1	52.8–55.3	56.7	56.4–57	< 0.001	0.316
Social functioning	51.8	50.5–53.1	55.2	55. - 55.4	< 0.001	0.470
Role-emotional	50.9	49.5–52.2	54.7	54.6–54.9	< 0.001	0.530
Mental health	52.1	50.8–53.4	56.3	56.1–56.6	< 0.001	0.514

### Prevalence of CAM use

Table 3 presents the weighted prevalence of past-year use of various CAM types among Singaporean residents by ethnicity. Overall, 6.4% of Singaporean adults reported having used at least one CAM modality in the past 12 months specifically for a mental illness. Among CAM users within each ethnic group, Chinese used CAM the least (5.9%), followed by Indians (7.6%) and Malays (8.1%). Chi-square analyses revealed statistically significant differences among the different ethnicities in terms of CAM use (*p* = 0.008). The five most commonly used types of CAM were prayer or other spiritual practices, with 2.7% of the population using it, followed by exercise and movement therapy (1.9%), massage therapy (1.6%), relaxation or meditation techniques (1.5%) and acupuncture (0.6%). Among these CAM types, only prayer and other spiritual practices showed statistical differences in usage among the different ethnicities.

**Table 3:** Prevalence of 12-month CAM use for mental illness in Singapore by ethnicity

	Chinese		Malay		Indian		Total		
	Weighted %	95% CI	Weighted %	95% CI	Weighted %	95% CI	Weighted %	95% CI	*p*
Overall, any CAM use	5.9	4.9–7.1	8.1	6.9–9.6	7.6	6.4–9.0	6.4	5.6–7.3	0.008
Prayer or other spiritual practices	2.0	1.4–2.8	5.5	4.5–6.7	4.2	3.3–5.3	2.7	2.2–3.3	< 0.001
Exercise or movement therapy	1.9	1.3–2.6	1.6	1.1–2.3	2.0	1.4–2.8	1.9	1.4–2.4	0.769
Massage therapy	1.6	1.1–2.3	1.9	1.3–2.7	1.7	1.2–2.4	1.6	1.2–2.1	0.786
Relaxation or meditation techniques	1.5	1.0–2.2	0.8	0.4–1.3	2.1	1.5–2.9	1.5	1.1–2.0	0.071
Acupuncture	0.6	0.3–1.1	0.5	0.3–1.0	0.5	0.3–0.9	0.6	0.4–1.0	0.835
Special diets	0.3	0.1–0.7	0.5	0.2–1.0	0.2	0.1–0.6	0.3	0.1–0.6	0.528
Any other non-traditional remedy or therapy	0.3	0.1–0.7	0.1	0.0–0.5	0.1	0.0–0.6	0.2	0.1–0.5	0.331
Spiritual healing by others	0.2	0.1–0.6	0.3	0.1–0.8	0.5	0.3–1.0	0.2	0.1–0.5	0.323
Chiropractic	0.2	0.1–0.5	0.2	0.1–0.6	0.1	0.03–0.5	0.2	0.1–0.4	0.607
Herbal therapy	0.1	0.03–0.5	0.2	0.1–0.5	0.03	0.004–0.2	0.1	0.05–0.3	0.152
High dose mega-vitamins	0.1	0.03–0.5	0.05	0.01–0.3	0	NA	0.1	0.03–0.4	0.587
Homeopathy	0	NA	0.2	0.1–0.5	0.2	0.05–0.6	0.1	0.03–0.1	< 0.001
Biofeedback	0.1	0.01–0.4	0	NA	0	NA	0.04	0.01–0.3	0.571
Energy healing	0	NA	0.1	0.03–0.5	0.1	0.03–.5	0.04	0.02–0.1	< 0.001
Hypnosis	0	NA	0.1	0.01–0.4	0	NA	0.007	0.001–0.05	0.008

### Factors associated with CAM use

Logistic regression was done for each of the three ethnic groups to estimate the odds of any CAM use (as shown in Table 4). Among the Chinese, females and those who never married were more likely to use CAM in the past 12 months (OR = 2, 95% CI = 1.25–3.20, *p* = 0.004; OR = 1.88, 95% CI = 1.08–3.28, *p* = 0.025 respectively). Unemployment was associated with higher odds of CAM use as well (OR = 2.78, 95% CI = 1.3–5.98, *p* = 0.009). Finally, those who met the criteria for a mental disorder had 3.74 times higher odds of using CAM than those who did not (95% CI = 2.28–6.15, *p* < 0.001). Age, education and income level were not found to be associated with CAM use.

**Table 4:** Odds of 12-month CAM use in Singapore by ethnicity

	Chinese			Malay			Indian		
	OR	95% CI	*p*	OR	95% CI	*p*	OR	95% CI	*p*
Age									
18–34 (reference)									
35–49	1.63	0.89–2.98	0.111	1.04	0.59–1.82	0.901	1.28	0.70–2.35	0.426
50–64	1.09	0.50–2.37	0.210	0.83	0.45–1.52	0.543	1.21	0.60–2.45	0.591
65+	1.22	0.36–4.15	0.748	0.18	0.05–0.66	0.010	0.76	0.30–1.90	0.554
Gender									
Male (reference)									
Female	2.00	1.25–3.20	0.004	1.50	0.99–2.26	0.054	2.21	1.42–3.45	< 0.001
Marital status									
Married (reference)									
Never married	1.88	1.08–3.28	0.025	1.96	1.15–3.32	0.013	3.11	1.74–5.55	< 0.001
Divorced/separated	0.28	0.06–1.24	0.093	1.51	0.71–3.20	0.284	2.36	1.13–4.90	0.022
Widowed	1.88	0.43–8.17	0.400	4.84	1.63–14.34	0.004	0.93	0.31–2.79	0.890
Education									
University (reference)									
Diploma	1.22	0.70–2.12	0.479	0.49	0.24–0.98	0.044	1.37	0.77–2.44	0.282
Vocational/ITE	0.61	0.18–2.09	0.427	0.45	0.21–0.94	0.034	1.47	0.57–3.82	0.423
Pre-U/JC	1.14	0.45–2.89	0.777	0.33	0.10–1.09	0.069	1.33	0.50–3.49	0.568
Secondary	0.83	0.41–1.68	0.609	0.38	0.18–0.79	0.009	1.27	0.64–2.53	0.495
Pri and below	0.27	0.06–1.17	0.081	0.40	0.16–0.99	0.047	0.67	0.25–1.81	0.435
Employment									
Employed (reference)									
Economically inactive	0.59	0.28–1.21	0.148	1.24	0.74–2.05	0.414	1.11	0.67–1.81	0.691
Unemployed	2.78	1.30–5.98	0.009	1.10	0.48–2.48	0.828	2.19	1.11–4.31	0.024
Monthly Household Income									
10,000 and above (reference)									
6000–9999	1.10	0.60–2.01	0.750	1.49	0.56–3.94	0.423	0.57	0.32–1.04	0.065
4000–5999	0.66	0.32–1.35	0.253	1.19	0.45–3.14	0.724	0.29	0.14–0.60	0.001
2000–3999	1.25	0.61–2.59	0.543	1.33	0.52–3.38	0.549	0.30	0.15–0.62	0.001
Below 2000	0.63	0.26–1.55	0.317	1.43	0.52–3.91	0.490	0.59	0.26–1.35	0.212
Any mental disorder									
No (reference)									
Yes	3.74	2.28–6.15	< 0.001	4.74	3.13–7.19	< 0.001	3.66	2.37–5.65	< 0.001

Among the Malays, older adults (65 years and above) were found to have lower odds (OR = 0.18, 95% CI = 0.05–0.66, *p* = 0.01) of using CAM compared to young adults (18 to 34 years). Compared with those who were married, higher odds were associated with people who never married (OR = 1.96, 95% CI = 1.15–3.32, *p* = 0.013) and people who were widowed (OR = 4.84, 95% CI = 1.63–14.3, *p* = 0.004). Higher educational level was also found to be significantly associated with greater CAM use. Similar to the Chinese, Malays who fulfilled the criteria for a mental illness diagnosis had higher odds of using CAM within the past year (OR = 4.74, 95% CI = 3.13–7.19, *p* < 0.001). Gender, employment and income were not found to be associated with CAM use.

Among the Indians, females were twice as likely to go for CAM treatment as opposed to males (95% = 1.42–3.45, *p* < 0.001), like the Chinese. Those who were single and divorced (or separated) were more likely to use CAM compared to those who were married (OR = 3.11, 95% CI = 1.74–5.55, *p* < 0.001; OR = 2.36, 95% CI = 1.13–4.9, *p* = 0.022 respectively). Unemployment and lower income were also associated with greater use of CAM. Like the other two ethnic groups, Indians who fulfilled the criteria for a mental disorder had higher odds of using CAM (OR = 3.66, 95% CI = 2.37–5.65, *p* < 0.001).

### CAM use and health-related quality of life

Table 5 presents the associations between self-reported health, as measured by SF-12 scores, and 12-month CAM use among the three ethnic groups, through separate linear regression analyses. After controlling for other sociodemographic variables, CAM use was found to significantly predict lower physical component scores for Chinese (B = − 1.35, 95% CI = − 2.67 – − 0.04, *p* = 0.044) and Indians (B = − 1.57, 95% CI = − 2.92 – − 0.21, *p* = 0.024), but not for Malays (B = − 0.82, 95% CI = − 1.9 – 0.25, *p* = 0.132). There were stronger associations between mental component scores and CAM use among all three ethnicities, with CAM use significantly predicting lower mental component scores for Chinese (B = − 2.94, 95% CI = − 4.69 – − 1.2, *p* = 0.001), Malays (B = − 3.4, 95% CI = − 4.88 – − 1.93, *p* < 0.001) and Indians (B = − 2.17, 95% CI = − 3.7 – − 0.65, *p* = 0.005).

**Table 5:** Regression results of 12-month CAM use as the predictor of SF-12 scores, by ethnicity

	Chinese			Malay			Indian		
	*β*	95% CI	*p*	*β*	95% CI	*p*	*β*	95% CI	*p*
Physical component score (PCS-12)	−1.35	−2.67--0.04	0.044	−0.82	−1.90-0.25	0.132	−1.57	−2.92--0.21	0.024
Physical functioning	−0.95	−2.27-0.37	0.16	−0.75	−1.83-0.32	0.171	−1.12	−2.43-0.19	0.094
Role-physical	−1.76	−3.08--0.45	0.008	− 2.30	−3.51--1.08	< 0.001	− 1.86	−3.33--0.39	0.013
Bodily pain	−2.99	−4.71--1.27	0.001	−1.54	−2.87--0.21	0.023	−2.86	−4.70--1.03	0.002
General health	−1.62	−3.62-0.37	0.11	−2.56	−4.26--0.87	0.003	−1.74	−3.54-0.06	0.059
Mental component score (MCS-12)	−2.94	−4.69--1.20	0.001	−3.40	− 4.88--1.93	< 0.001	−2.17	− 3.70--0.65	0.005
Vitality	−2.14	−3.88--0.40	0.016	−1.18	−2.78-0.42	0.148	−2.22	−3.86--0.59	0.008
Social functioning	−3.06	−4.74--1.37	< 0.001	−2.04	−3.20--0.87	0.001	−1.14	−2.55-0.27	0.113
Role-emotional	−3.14	−4.83--1.45	< 0.001	−3.06	−4.56--1.56	< 0.001	−1.66	− 3.10--0.21	0.025
Mental health	−2.07	−3.84--0.30	0.022	−3.90	−5.45--2.35	< 0.001	−2.91	−4.50--1.32	< 0.001

Models controlled for gender, age, marital status, education, employment, income and mental disorder diagnosis (Y/N)

## Discussion

To the best of our knowledge, this is the first study that has examined characteristics of CAM use for mental illness treatment and its association with health-related quality of life among the major ethnic groups in Singapore. The weighted prevalence of past 12-month CAM use for a mental illness in Singapore’s adult resident population was found to be 6.4%; among those who met the criteria for a mental disorder in their lifetime, 17% had used CAM in the same period. This is about four times the proportion of CAM users who had no mental disorder. While the relatively higher proportion of CAM users among those who met the criteria for a mental disorder (compared with those who did not) is consistent with other studies which found the same pattern, the rates of CAM use found in this study differed from those found in other studies [9, 18, 30]. This may be attributed to the different definitions and criteria used pertaining to CAM use and the mental disorders identified in the studies. For example, the percentage of CAM users that was found in this study among people with a mental disorder was much lower than that found in a previous study on CAM use and mental disorders in Singaporean older adults, which reported a prevalence rate of 53% [18]. This might be due to the difference in scope of CAM use investigated between the two studies – the current study asked specifically about CAM use for mental illness treatment, while the other study did not, and merely examined CAM use in general. On the other hand, the operationalization of CAM use in de Jonge et al.’s study as contact with a CAM service provider, as opposed to the current study’s inclusion of practitioner- and self-directed usage, may explain the current study’s higher reported rates of CAM users among those with a 12-month mental disorder, compared to what was found in de Jonge et al.’s study [9].

Analyses further revealed that across all ethnicities, having a mental disorder increased the odds of using CAM by at least 3.6 times, a finding similarly observed in a recent US study [8]. A possible explanation for this increased use is that compared to conventional clinical health services for mental health, CAM therapies are more easily accessible, and the use of CAM for mental illness is associated with less stigma as well [31]. Another possible reason could be that the cost of CAM is relatively lower than that of conventional healthcare [32].

These findings have certain implications on the delivery of health care to people with mental illness in clinical settings. Conventional health providers may find it prudent to address whether their patients are also using CAM, and tailor their treatment programs accordingly. On the other hand, it is also important for CAM practitioners to be aware that their clients may have mental illnesses that clinicians and specialists are more equipped to address. Ultimately, the quality of mental health care and illness management can be facilitated by improved coordination between conventional mental health specialists and CAM practitioners, to provide more holistic treatment plans for patients with mental illness [33].

Among the ethnicities, CAM was found to be used most by Malays, followed by Indians, and lastly Chinese. Among the five most commonly used forms of CAM, only the use of prayer or other spiritual practices differed among the three ethnic groups, with Malays having the highest proportion of users, followed by Indians and Chinese. This increased use may reflect the strong religious and spiritual beliefs of Malays, who are predominantly Muslims, and the important role that religion plays in their lives. The interplay between prayer and mental health, in the context of Muslims, can be seen in how the processes of prayer, reciting the Qur’an, and other spiritual practices are deeply inculcated as a means of resolving physical and mental hardships [34, 35]. These religious teachings, together with the high level of commitment of Malays in general to their religion, may account for the higher tendency of Malays to turn to prayer and other spiritual practices for addressing their mental illness, as compared to people from other ethnicities, who are more heterogeneous in their religious and spiritual beliefs [36]. As this is a preliminary finding, future studies can look at the association between ethnicity, religion and CAM use as a treatment for mental illness for a better understanding of the cultural mechanisms underpinning CAM use [37].

While the variables found in this study to be associated with CAM use corresponded with that found in other studies, significant differences were found in the patterns of these associations among the different ethnic groups. Among Malays, age and education were found to be significantly associated with CAM use. Consistent with other studies [6, 8], older Malays were found to have lower odds of using CAM in general. This may be a reflection of their perceptions of their own illness severity and attitudes towards help-seeking, as documented in a study which found stronger beliefs of self-reliance and greater passivity in older people when it came to addressing their mental illness symptoms [38]. With regards to education, a university education was found to be associated with higher odds of CAM use, a finding also corroborated by other studies [8, 17, 30, 39]. A plausible explanation for this may be that a higher education increases people’s awareness of CAM to treat mental illness, as well as their capacity to seek out CAM as an alternative treatment resource [17]. Additionally, a higher education level may be associated with increased health literacy [40], which is “the capacity to obtain, process, and understand basic health information and services needed to make appropriate health decisions” [41], and which has been shown to be linked with increased CAM use [42, 43]. However, these associations were only clearly found in the Malay group, and not in the Chinese or Indian groups. In line with other studies [44–46], we found increased use among the unemployed, and unemployment significantly increased the odds of turning to CAM among Chinese and Indians. It is possible that CAM may have been perceived as a cheaper alternative to more Westernized forms of treatment by this group. Gender was also found to be associated with CAM use among Chinese and Indians, with females having higher odds of 12-month CAM use, consistent with previous studies [47, 48]. This increased use may be attributed to the greater concern and care women tend to show towards their own health, compared to men, and their greater proactiveness in resolving health issues such as mental illness symptoms [49]. These associations were not found in Malays. Finally, lower income was only found to be associated with lower odds of CAM use in Indians, but not in Malays or Chinese. The only common finding among all three ethnic groups was that being unmarried significantly increased the odds of CAM use across all three groups, which is consistent with another study that found a higher likelihood for unmarried people to utilize mental health services than married people [50].

Similarities were identified in the association between CAM use and health status among the different ethnicities. After controlling for the other sociodemographic variables, CAM use was found to be associated with negative mental health-related quality of life across all ethnicities, in line with other studies which demonstrated an increased likelihood for CAM users to report poorer mental health [8, 18]. This association may be a reflection of the tendency for people with poorer mental health to seek CAM in a bid to manage and improve their mental well-being. While the exact causal relationship between CAM use and poor mental health cannot be explicitly established in the current study, this finding nevertheless espouses the need for greater awareness of CAM use as an indicator of mental illness, on the part of both CAM providers and conventional health service providers.

Several limitations should be considered when interpreting the findings of this study. Firstly, the administration of the survey as a self-report interview makes respondents’ answers susceptible to recall bias. Secondly, the lack of cultural specificity of the CIDI to the local Singaporean context may have led to underreporting of CAM use by respondents if they did not recognize that what they used for their mental illness (for example, traditional Chinese herbal medicines) was a type of CAM (in the CIDI, this fell under herbal therapy). This was minimized by interviewers’ probing for any other therapy or remedy used for mental illness and immediate clarification of any queries brought up by respondents regarding the use of alternative therapies. Thirdly, due to the cross-sectional design of the study, the exact causality of the relationship between CAM use and the sociodemographic characteristics, and that between CAM use and health-related quality of life, cannot be established. Finally, as the CIDI was not designed specifically to assess CAM use, an in-depth understanding of the frequency, duration, specific reasons and underlying mechanisms behind CAM use was not possible. Despite these limitations, this study provides insight into ethnic differences in the use of CAM for mental illness, via discrete sub-group analyses of CAM use associations with the different ethnic groups in Singapore. The study also demonstrates that factors relating to CAM use do vary among different ethnicities, with certain factors being of more importance to people of a given ethnic group when considering CAM for the treatment of mental illness.

## Conclusion

This study set out to investigate the differences in the use of CAM to treat mental illness among the different ethnic groups in Singapore. Analyses revealed several differences in the associations between CAM use and sociodemographic variables regarding the use of CAM, reflecting variations in how and when CAM is utilized by people of different ethnicities. CAM was used most by Malays, followed by Indians and lastly Chinese. Age and education were significantly associated with CAM use among Malays, but not among Indians or Chinese. On the other hand, gender and employment status were associated with CAM use among Indians and Chinese, but not Malays. Finally, the association between income and CAM use was found only for Indians, but not for Chinese and Malays. Despite these differences, having a mental illness significantly increased CAM use in Singapore regardless of ethnicity. This highlights the need for communication and collaboration between conventional and CAM providers to facilitate detection, planning and treatment of mental illness.

## Data Availability

The datasets used and/or analyzed during the current study are available from the corresponding author on reasonable request.

## Abbreviations

CAM: complementary and alternative medicine
DSM-IV: Diagnostic and Statistical Manual of Mental Disorders, Fourth Edition
SF-12: Short Form-12
SMHS: Singapore Mental Health Study
WHO: World Health Organisation
WMH CIDI: World Mental Health Composite International Diagnostic Interview
